# Dynamic of Pediatric Invasive Bacterial Infections in Switzerland: A 2017–2024 Time-series Analysis of National Data

**DOI:** 10.1097/INF.0000000000004886

**Published:** 2025-06-05

**Authors:** Manon Jaboyedoff, Anita Niederer-Loher, Christian R. Kahlert, Pierre Alex Crisinel, François Angoulvant

**Affiliations:** From the * Department Women-Mother-Child, Unit of Pediatric Infectious Diseases and Vaccinology, Service of Pediatrics, Lausanne University Hospital and University of Lausanne, Lausanne, Switzerland; † Division of Infectious Diseases and Hospital Epidemiology, Children’s Hospital of Eastern Switzerland, St Gallen, Switzerland; ‡ Department Women-Mother-Child, Service of Pediatrics, Lausanne University Hospital and University of Lausanne, Lausanne, Switzerland.

**Keywords:** *Streptococcus pyogenes*, group A streptococcus, *Neisseria meningitidis*, *Streptococcus pneumoniae*, *Haemophilus influenzae*

## Abstract

This nationwide, population-based, time-series analysis of invasive bacterial infections (IBIs) in Swiss children shows that vaccine-preventable IBIs were less affected by nonpharmaceutical interventions during the coronavirus disease 2019 (COVID-19) pandemic compared with invasive group A streptococcal infections. These findings emphasize the distinct epidemiological dynamics of bacterial pathogens in response to public health measures.

During the first winter following the lifting of nonpharmaceutical interventions (NPIs) against COVID-19, the incidence of pediatric community-acquired infections, including invasive bacterial infections (IBIs), increased beyond that of the prepandemic years.^1^ The incidence of invasive group A streptococcal infection (iGAS), invasive pneumococcal disease (IPD) and invasive meningococcal disease (IMD) increased during the first post-NPIs year.^2–4^ This happened after a period of lower incidence of IBIs, while NPIs were in place.^5,6^

*Streptococcus pneumoniae*, *Streptococcus pyogenes, Neisseria meningitidis* and *Haemophilus influenzae* are common causes of IBIs in children. These bacteria colonize the oropharynx and are transmitted by droplets.

We aimed to assess how the incidence of IPD, IMD, invasive *H. influenzae* disease (IHD) and iGAS in Swiss children varied after NPIs implementation and during the first 2 years following lifting of NPIs.

## METHODS

### Study Design

We conducted a population-based interrupted time-series analysis over 76 months (November 1, 2017, to February 29, 2024). We defined 4 time periods: A preintervention period, before the COVID-19 pandemic and NPIs effects, and 3 postintervention periods (during NPIs effects, the first postpandemic year and the second postpandemic year). We analyzed the incidence of 4 bacterial infections capable of human-to-human transmission: IPD, IMD, IHD and iGAS. In Switzerland, NPIs were first implemented on March 16, 2020. Until the normal situation was declared again on April 1, 2022, various NPIs were implemented at different time points such as restrictions on gatherings, public space closure and wearing of masks.^7^

### Population and Case Definition

The population of interest consisted of all children and adolescents 0–16 years old and living in Switzerland during the study period, totaling about 1.5 million individuals. iGAS was defined as the isolation of *S. pyogenes* from a normally sterile site or the presence of a toxic shock syndrome or necrotizing fasciitis and *S. pyogenes* isolation from a nonsterile site. IPD, IMD and IHD were defined as the isolation of *S. pneumoniae*, *N. meningitidis* or *H. influenzae* from a normally sterile site.

### Outcomes

The main outcomes were the IPD, IMD, IHD and iGAS monthly incidences per 100,000 children under 17 years of age. The secondary outcome was the pooled incidence of vaccine-preventable IBI, combining IPD, IMD and IHD.

### Data Source

We used 2 data sources. First, we used data from the mandatory reporting of IPD, IMD and IHD from the Swiss Federal Office of Public Health. All doctors, hospitals, healthcare institutions and laboratories in Switzerland have the legal obligation to report certain communicable diseases, including those caused by *S. pneumoniae*, *N. meningitidis* and *H. influenzae*. Second, we used data from the reporting of iGAS to Swiss Pediatric Surveillance Unit (SPSU). The SPSU functions as a monitoring system specifically designed to track occurrences of rare pediatric diseases or uncommon complications arising from more prevalent illnesses among hospitalized children in Switzerland. Participation in this surveillance involves all 29 pediatric clinics across Switzerland, each actively reporting cases monthly. iGAS are reported since November 2017.

### Statistical Analysis

To calculate the incidence of IBI per 100,000 children, we used age-specific Swiss population data provided by the Swiss Federal Statistical Office.^8^ We defined 4 time periods to analyze the impact of NPIs: (1) preintervention period, 30 months before implementation of NPIs for COVID-19 (November 1, 2017, to March 31, 2020), (2) first postintervention period, from the start to the end of NPIs (24 months, from April 1, 2020, to March 31, 2022), (3) second postintervention period, the first year post-NPIs (12 months, from April 1, 2022, to March 31, 2023) and (4) third postintervention period, the second year post-NPIs (10 months, from April 1, 2023, to February 29, 2024). We estimated the impact of NPIs implementation and NPI lifting on the incidence of IBIs using quasi-Poisson regression modeling, accounting for seasonality. The time unit was 1 month. We used data collected before April 2020 to generate a model fitting the observed absolute incidence of each IBI and predict the expected IBIs incidence if NPIs had never been implemented.

### Ethics

Mandatory reporting of IPD, IHD and IMD to the Swiss Federal Office of Public Health is performed in accordance with the Federal Department of Home Affairs Ordinance of December 1, 2015, on the reporting of observations of communicable diseases in humans (SR 818.101.126, https://www.fedlex.admin.ch/eli/cc/2015/892/fr). Reporting of iGAS to the SPSU was determined to not require formal ethical approval by the St. Gallen ethical committee (BASEC Nr. Req-2023-00750) as collected data are anonymous.

## RESULTS

**FIGURE 1.**
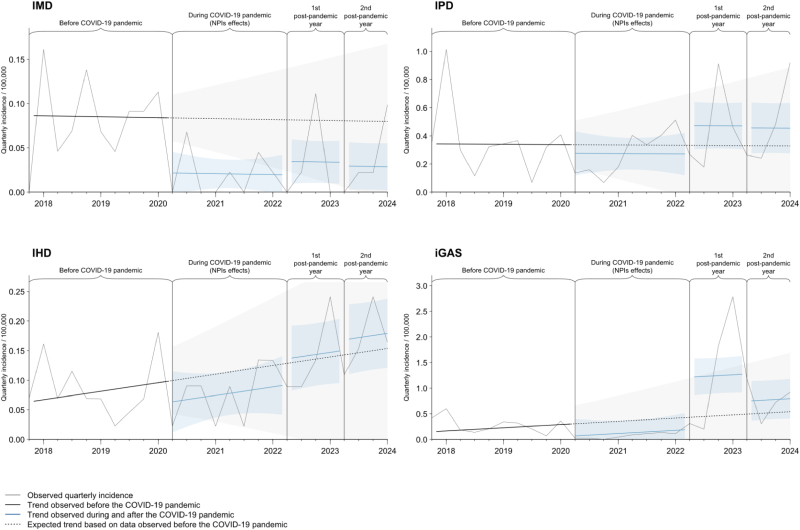
Quarterly incidence of invasive meningococcal (IMD), pneumococcal (IPD), *H. influenzae* (IHD) and group A streptococcal (iGAS), diseases in Swiss children 0–16 years of age. The gray lines show the observed data. The black line shows the deseasonalized trend based on observed data before the NPIs implementation. The blue lines show the deseasonalized trend based on observed data after NPIs implementation, with blue shaded area showing the 95% confidence intervals. The black dashed lines show the expected deseasonalized trend if nonpharmaceutical interventions (NPIs) were not implemented and then lifted, based on data from November 2017 to March 2020. The vertical lines show the limit between periods (pre-NPIs, during NPIs, first postpandemic year and second postpandemic year).

Between November 2017 and February 2024, a total of 405 IPD, 54 IMD, 117 IHD and 503 iGAS were declared in children 0–16 years old (Fig. 1).

While NPIs were in place, the incidence of IMD decreased significantly [−78%, 95% confidence interval (CI): −95% to −12%, *P* = 0.032], as the incidence of iGAS (−68%, 95% CI: −89% to −10%, *P* = 0.031). There was no statistically significant decrease in IPD and IHD incidence.

During the first postpandemic year (2022/2023), there was a significant rise in the incidence of iGAS, which increased by about fourfold compared to the pre-NPIs period (419%, 95% CI: 26%–2043%, *P* = 0.023). During the second postpandemic winter (2023/2024), the incidence of iGAS was not significantly increased (187%, 95% CI: −51% to 1578%, *P* = 0.24). The incidence of IPD, IMD and IHD remained unchanged during both postpandemic periods.

The analysis of the combined incidence of vaccine-preventable IBI (IPD, IMD and IHD) showed no significant change during the pandemic and postpandemic periods compared to the pre-NPI period.

## DISCUSSION

In this time-series analysis of pediatric IBIs in Switzerland, we found that NPIs applied during the COVID-19 pandemic influenced the pattern of pediatric IBIs with a decrease in IMD and in iGAS following the implementation of NPIs and a significant increase in iGAS the first winter after their lifting. The incidence of iGAS was not significantly increased in the second winter (2023/2024) following NPI lifting.

The increase in the incidence of iGAS during the first postpandemic year (2022/2023), its decrease during the second postpandemic winter (2023/2024) and the absence of an increased incidence of that magnitude of IMD, IPD and IHD, which are vaccine-preventable diseases, reinforce one hypothesis that this increase was in part due to reduced population immunity to group A streptococcus (GAS).^9^ The Swiss vaccination schedule for infants indeed include immunization for IPD (pneumococcal conjugate vaccine 13), IMD (serogroups A, C, W and Y since 2019, and since 2024 serogroup B) and IHD (type B) (Swiss Vaccination Schedule, https://www.bag.admin.ch/). The vaccine coverage in 2022 in children 2 years old was 93% for IPD and 82% for IMD (ACWY), and 95% for IHD in those 3 years of age.^10^ Around 50% of IMDs were due to *N. meningitidis* serogroup B before 2023 in Switzerland. As no vaccine recommendation against *N. meningitidis* serogroup B was in place before 2024, this could explain why NPIs had a more important impact on IMD than on IPD and IHD in Swiss children. The decline and absence of rebound in IMD in Switzerland might not only be explained by the effect of NPIs, but also by the implementation of the MenACWY vaccine in 2019. In addition to vaccination, the rise in viral respiratory infections during the first postpandemic winter also influenced the epidemiology of pediatric IBIs. In France, for example, while pneumococcal carriage remained stable, the magnitude of changes in IPD incidence was correlated with that of RSV and influenza.^11^ In this French population, the increase in incidence in IPD was, however, greater in children 5–17 years old than in children younger than 5 years, highlighting differences between recently vaccinated and nonrecently vaccinated age-groups. The reduced population immunity due to NPIs, therefore, likely played an important role in the resurgence of pediatric community-acquired infections and might explain why iGAS surged, but not vaccine-preventable IBIs—or at least not to the same magnitude. Rebounds in vaccine-preventable IBIs were observed in some countries following the lifting of NPIs, and in certain cases, this resurgence may have been associated with suboptimal vaccine coverage, suggesting that vaccination gaps could amplify the post-NPI reemergence of these infections.^12^ By maintaining an optimal vaccination coverage for vaccine-preventable IBIs like IPD, IMD and IHD, the postpandemic resurgence of these infections could likely have been mitigated.

### Strengths and Limitations

The strengths of this study are the quality of the data, covering an entire country and reporting incidence of IBIs before the pandemic and up to winter 2023/2024. Our data were unlikely influenced by fluctuations in the number of reports during the pandemic as it is a legal requirement to report IHD, IMD and IPD and that iGAS are reported through a well-established surveillance system. The main limitations are that although we used national data, IBIs in children are rare events, additionally limited by the small size of the population of Swiss children. As a result, minor variations in the incidence of IBIs may not have been captured. Moreover, IHD encompasses all invasive *H. influenzae* infections, including nontypeable *H. influenzae*, which are not the main cause of IHD in children and are not the target of *H. influenzae* vaccination.

## CONCLUSIONS

While lifting COVID-19-related NPI initially led to a major surge in iGAS among Swiss children, our study shows that incidence rates returned mostly to baseline within a year. The incidences of vaccine-preventable IBIs were less affected by NPIs, suggesting that population immunity plays an important role in shaping the epidemiology of these infections. In Switzerland, high vaccination coverage likely contributed to the sustained low incidence of vaccine-preventable IBIs. These findings highlight the importance of maintaining high vaccine uptake.

## ACKNOWLEDGMENTS

We thank Hanna Hildenbrand and Ornella Luminati from the Swiss Federal Office of Public Health for their help in data acquisition.
